# Ivermectin-induced gene expression changes in adult *Parascaris univalens* and *Caenorhabditis elegans*: a comparative approach to study anthelminthic metabolism and resistance in vitro

**DOI:** 10.1186/s13071-022-05260-4

**Published:** 2022-05-05

**Authors:** Faruk Dube, Andrea Hinas, Shweta Roy, Frida Martin, Magnus Åbrink, Staffan Svärd, Eva Tydén

**Affiliations:** 1 Division of Parasitology, Department of Biomedical Sciences and Veterinary Public Health, Swedish University of Agricultural Sciences, Box 7036, 750 07 Uppsala, Sweden; 2 Department of Cell and Molecular Biology, Uppsala University, 751 24 Uppsala, Sweden; 3 Section of Immunology, Department of Biomedical Sciences and Veterinary Public Health, Swedish University of Agricultural Sciences, Box 7036, 750 07 Uppsala, Sweden

**Keywords:** Equine roundworm, Gene expression, Anthelmintic resistance, RNA sequencing

## Abstract

**Background:**

The nematode *Parascaris univalens* is one of the most prevalent parasitic pathogens infecting horses but anthelmintic resistance undermines treatment approaches. The molecular mechanisms underlying drug activity and resistance remain poorly understood in this parasite since experimental in vitro models are lacking. The aim of this study was to evaluate the use of *Caenorhabditis elegans* as a model for *P. univalens* drug metabolism/resistance studies by a comparative gene expression approach after in vitro exposure to the anthelmintic drug ivermectin (IVM).

**Methods:**

Twelve adult *P. univalens* worms in groups of three were exposed to ivermectin (IVM, 10^–13^ M, 10^–11^ M, 10^–9^ M) or left unexposed for 24 h at 37 °C, and total RNA, extracted from the anterior end of the worms, was sequenced using Illumina NovaSeq. Differentially expressed genes (DEGs) involved in metabolism, transportation, or gene expression with annotated *Caernorhabditis elegans* orthologues were identified as candidate genes to be involved in IVM metabolism/resistance. Similarly, groups of 300 adult *C. elegans* worms were exposed to IVM (10^–9^ M, 10^–8^ M and 10^–7^ M) or left unexposed for 4 h at 20 °C. Quantitative RT-PCR of RNA extracted from the *C. elegans* worm pools was used to compare against the expression of selected *P. univalens* candidate genes after drug treatment.

**Results:**

After IVM exposure, 1085 DEGs were found in adult *P. univalens* worms but the relative gene expression changes were small and large variabilities were found between different worms. Fifteen of the DEGs were chosen for further characterization in *C. elegans* after comparative bioinformatics analyses. Candidate genes, including the putative drug target *lgc-37*, responded to IVM in *P. univalens*, but marginal to no responses were observed in *C. elegans* despite dose-dependent behavioral effects observed in *C. elegans* after IVM exposure. Thus, the overlap in IVM-induced gene expression in this small set of genes was minor in adult worms of the two nematode species.

**Conclusion:**

This is the first time to our knowledge that a comparative gene expression approach has evaluated *C. elegans* as a model to understand IVM metabolism/resistance in *P. univalens*. Genes in *P. univalens* adults that responded to IVM treatment were identified. However, identifying conserved genes in *P. univalens* and *C. elegans* involved in IVM metabolism/resistance by comparing gene expression of candidate genes proved challenging. The approach appears promising but was limited by the number of genes studied (*n* = 15). Future studies comparing a larger number of genes between the two species may result in identification of additional candidate genes involved in drug metabolism and/or resistance.

**Graphical Abstract:**

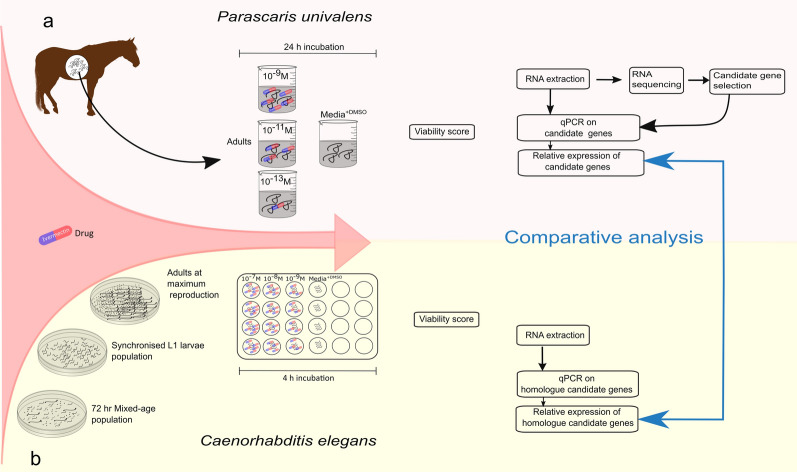

**Supplementary Information:**

The online version contains supplementary material available at 10.1186/s13071-022-05260-4.

## Introduction

The equine roundworm, *Parascaris univalens*, is one of the most important pathogenic parasites infecting foals, with a prevalence of 31–58% on stud farms [1–4]. Clinical manifestations of infection include stunted growth, respiratory symptoms, and in severe cases lethal obstruction and rupture of the small intestines [5]. In addition, *P. univalens* are known shedders of high numbers of eggs [6], and larvated eggs can remain viable on grazing fields for many years [7]. This asserts a high infection pressure on farms, resulting in foals requiring several treatments with deworming drugs during their first year [8].

Three main classes of anthelmintic drugs, benzimindzoles (BZ), tetrahydropyrimidines (THP), and macrocyclic lactones (MLs), are registered for use in horses to combat parasitic infections, including *Parascaris* spp. Macrocyclic lactones are the most commonly used drug class in veterinary medicine due to their high efficacy, low toxicity, and broad spectrum of target parasites [9]. The primary target of MLs in parasitic nematodes are glutamate-gated ion channels (GluCls), where for example the ML drug ivermectin (IVM) binds irreversibly to cause hyperpolarization of the pharynx and flaccid paralysis of muscles in the worm [10]. Development of anthelmintic resistance (AR) in *Parascaris* spp. has evolved as a result of extensive drug usage [11], and resistance to MLs is now widespread [3, 4, 11–14].

Several ML drug resistance mechanisms have been proposed, e.g. polymorphisms or changes in expression of the GluCls target and transport protein genes (see [15] for review). In addition, increased expression of genes encoding drug-metabolizing enzymes has been shown to be involved in resistance [16]. Polymorphisms cause conformational changes and alter drug-binding sites in the GluCl targets in the pathogenic nematodes *Cooperia oncophora* [17] and *Haemonchus contortus* [18]. In addition, decreased expression of drug target genes may lead to a reduction of drug-binding sites and thus reduced drug effectiveness [19, 20]. Transport protein genes, particularly encoding P*-*glycoproteins (Pgps) and other ATP*-*binding-cassette family members, have been implicated in anthelmintic drug resistance through the elimination of xenobiotic substances from the cell, thus preventing the drugs from reaching their target sites [21–24]. Multiple studies have also reported higher constitutive expressions of Pgps in resistant parasite strains compared to their susceptible counterparts [20, 21]. The metabolism of drugs is biphasic. In the first phase (Phase I), the drug is converted by oxidation, reduction, or hydrolysis to a more reactive compound that can be conjugated with an endogenous molecule such as glutathione or glucose in the second phase (Phase II). As a result, a soluble, inactive drug is generated that can be expelled from the cell [25]. Increased constitutive expression of Phase I [26] and Phase II metabolic genes [27] was reported in resistant *H. contortus* isolates after in vitro exposure with BZs. However, the exact roles of Phase I and II enzymes in development of ML resistance in parasitic nematodes remain unknown.

The genetic mechanisms underlying ML resistance in *P. univalens* are inadequately understood, and further research is hampered by the complex host-dependent life cycle. Moreover, in vivo studies using the parasite host would be more comprehensive, but are challenged by both ethical and financial constraints. Previous studies have only been conducted in an in vitro setting using live adult worms isolated from slaughtered horses or euthanized foals from research herds [28–30]. However, not only is this approach laborious and costly, but it is also inefficient because slaughter of foals is rare and the number of research herds is limited. In addition, the unnatural in vitro culture environment has been reported to have undesirable effect on gene expression of *P. univalens* [29]. Therefore, there is a need for the development of in vitro experimental models for *P. univalens*. Recently, the larval stage of *P. univalens* has been explored as a possible in vitro model, but authors reported variation in gene expression between larvae and adult *P. univalens* [31]. The free-living nematode *Caenorhabditis elegans* has been used as an in vitro model for parasitic nematodes, particularly those belonging to the same taxonomic clade, V, such as *H. contortus* [32–35]. Although *P. univalens* belongs to a different clade, III, previous studies have employed transgenic lines of *C. elegans* as models for interpretation of ML-resistance in *P. univalens* [28, 36, 37]. Factors that support *C. elegans* as a viable model for parasitic nematodes include possession of similar drug targets [38, 39] and being cheap and easy to maintain in the laboratory. In addition, *C. elegans* has a short life cycle of 3.5 days and a plethora of powerful genetic tools for manipulation are readily available [40]. To improve the likelihood of deriving conclusions from *P. univalens* research that uses *C. elegans* as a model organism, it is necessary to assess its applicability, at the very least in terms of gene expression following xenobiotic exposure.

In the current study, we investigated differentially expressed genes (DEGs), which encode drug targets, transporters, transcription regulators, or enzymes involved in drug metabolism in adult *P. univalens* after in vitro exposure to the ML substance, IVM. We further investigated the expression profile of the *C. elegans* orthologues of the above genes in adult *C. elegans* after in vitro exposure to IVM. Our objective was to evaluate whether *C. elegans* is a suitable model for *P. univalens* by studying and comparing gene expression of selected candidate genes in *P. univalens* and *C. elegans* after in vitro IVM exposure. Our findings showed that candidate genes, including the putative drug target gene, *lgc-37*, responded to IVM in *P. univalens*, but marginal to no response was observed in *C. elegans* despite dose-dependent behavioral effects observed in *C. elegans* after IVM exposure.

## Materials and methods

### *Parascaris univalens*

**Fig. 1:**
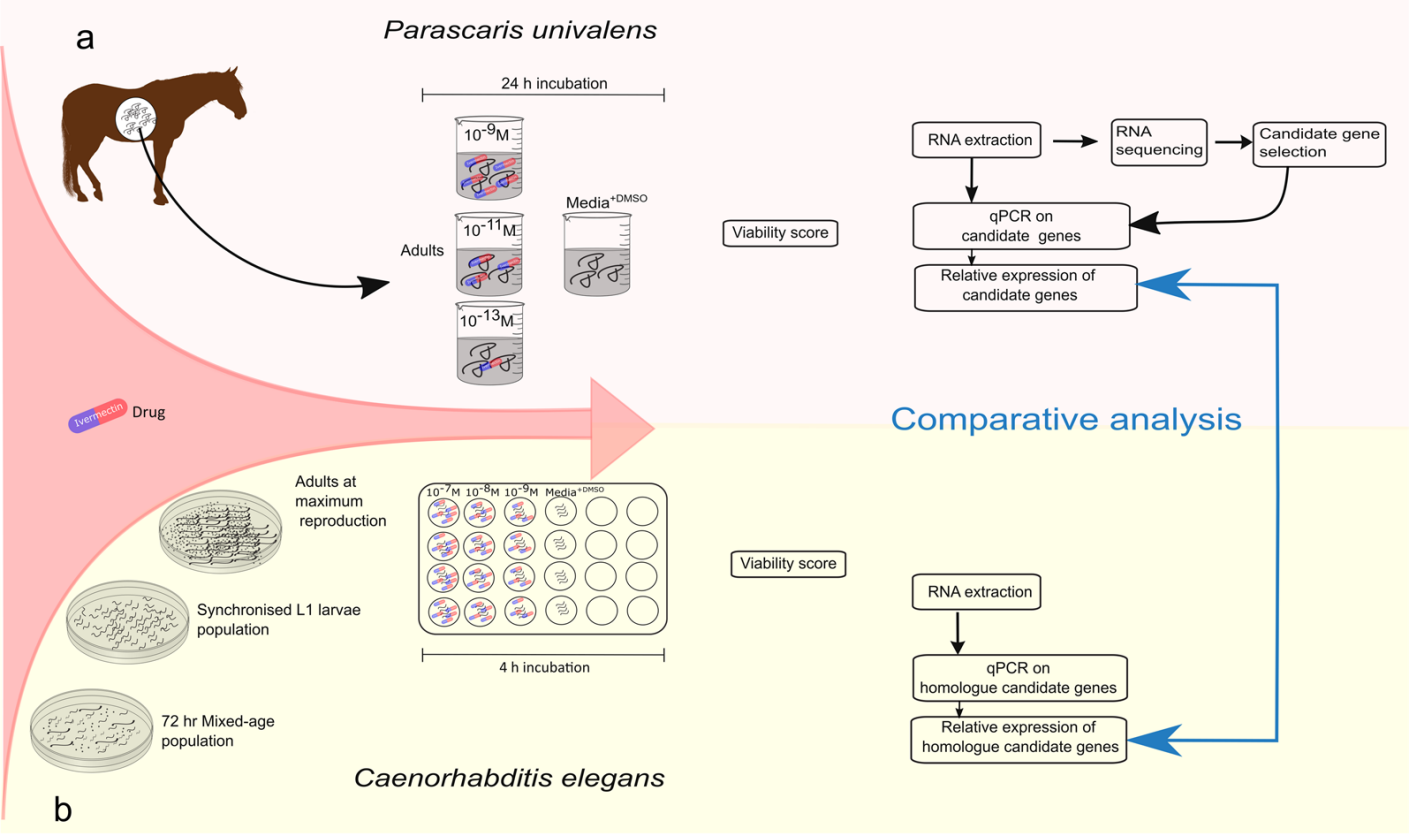
Illustration of the study design. Section **a** depicts experimental setup of *Parascaris univalens*. Adult worms were exposed to IVM drug concentrations, 10^–13^, 10^–11^, 10^–9^ M, and media^+DMSO^ (control). After viability scoring, RNA was sequenced, candidate genes selected, and quantitative RT-qPCR performed on the selected candidate genes. Section **b** depicts experimental setup of *Caenorhabditis elegans.* Worm pools of adults at maximum reproduction were exposed to IVM drug concentrations, 10^–9^, 10^–8^, 10^–7^ M, and media^+DMSO^ (control). Quantitative RT-PCR was performed on the *P. univalens* orthologues of selected candidate genes. Relative gene expression of the candidates was compared between nematode species

Adult *P. univalens* worms collected from two anthelmintic-naïve Icelandic foals at an abattoir in Selfoss, Iceland, were exposed to IVM in vitro. A detailed account of the experimental setup has been described in Martin et al. [29]. Care was taken to choose only the largest worms (i.e. females) for the study. Briefly, worms were incubated for 24 h in cell culture media containing 0.1% DMSO supplemented with 10^–13^, 10^–11^ or 10^–9^ M IVM. In addition, a control group (media^+DMSO^) of unexposed worms receiving cell culture media containing 0.1% DMSO was used (Fig. 1a). The experiment was performed in three biological replicates with at least three worms in each group. One worm from each subgroup was dissected. The anterior end (pharynx, nerve cell bodies (‘ganglia’), nerve ring, and part of the intestine) of the worm was cut into fine pieces and suspended in 1 ml Trizol (Invitrogen, Carlsbad, USA). After homogenization with a glass tissue grinder, chloroform was added and the aqueous phase of the homogenized suspension was isolated. The aqueous phase was advanced into NucleoSpin® RNA Plus Kit (Macherey Nagel, Düren, Germany) for RNA extraction according to the manufacturer’s instruction. Before preparing sequencing libraries from 500 ng total RNA, Fragment Analyzer (Agilent, Santa Clara, USA) was used to determine RNA concentration and quality. Illumina NovaSeq S1 flow cells and 100-bp paired end v1 sequencing chemicals were used to sequence three biological replicates per condition (Fig. 1a).

### RNA sequencing analysis

Reads were quality assessed using blastp [41] in a pipeline available at https://github.com/SLUBioinformaticsInfrastructure/RNAseq_nf. Mapping and quantification of reads against the predicted reference *P. univalens* transcriptome (https://parasite.wormbase.org/Parascaris_univalens_prjna386823/Info/Index/) were performed using Salmon v.0.11.3 [42]. Salmon output was matrix-summarized using R package tximport [43] for DESeq2 v.1.22.2 R package to determine DEGs as log_2_foldchange). Wald test derived *P*-values from DESeq2 were modified for multiple testing using the Benjamini-Hochberg approach-based p.adjust function in R base [44]. Differentially expressed genes were defined as those with an adjusted *P*-value < 0.05. Functional annotations of DEGs were identified by a BlastP search (*e*-value ≤ 10^−5^) of their respective protein sequences against the Swiss-Prot database.

To determine the number of genes shared by different drug concentrations, gene IDs were used to build comparative Venn diagrams in R v.3.6.1 using the venn.diagram function of the VennDiagram package [45]. The get.venn.partition function was used to retrieve gene IDs corresponding to each Venn diagram partition.

### Principal component analysis

To analyze differences in the response to IVM exposure among individual worms, principal component analysis (PCA) was employed using scaled read counts > 0 for 10,830 genes from each worm (*n* = 12). The prcomp function in R was used on the scaled read count to perform PCA. To determine how much variation each principal component (PC) accounts for in the data, standard deviations generated by PCA were used to compute variances. The percentage variances were visualized in a scree plot using barplot function in R. The top two PCs, which account for most variation in the data, were used to build a PCA plot using ggplot2 R package.

### Selection of candidate genes

The ten most DEGs in *P. univalens* were identified for each IVM concentration and considered as potential candidate genes. In addition, genes shared by two or more IVM concentrations were considered as potential candidates. Using the BiomaRt package in R, the potential candidate gene IDs were used to retrieve their respective ontologies and *C. elegans* orthologues from WormBase ParaSite (https://parasite.wormbase.org). From the pool of potential candidates, the selection of candidate genes was based on the presence of an orthologue gene in *C. elegans* and that they were functionally characterized as drug targets or involved in at least one of the following processes: xenobiotic metabolism, metabolite transportation, and gene expression regulation.

The similarity of *P. univalens* and corresponding *C. elegans* orthologues from WormBase were verified on the I-TASSER web-server [46] using their respective amino acid sequences.

### *Caenorhabditis elegans*

#### *Caenorhabditis elegans* culture and maintenance

Wild-type N2 Bristol strain of *C. elegans* obtained from Caenorhabditis Genetics Center (CGC) was used in this study.

Worms were grown and maintained on OP50 *Escherichia coli* seeded Nematode Growth Media (NGM) at 20 °C as per standard methods [47].

#### Worm synchronization

Unless otherwise stated all M9 buffer [48] used was supplemented with 0.005% Tween20 to avoid worms/embryos adhesion to plastic ware.

*Caenorhabditis elegans* of different life stages were cultured on 10-cm-diameter NGM agar plates for 72 h to gravid adults. Worms were harvested with ice-cold M9 buffer into 15-ml Falcon tubes and synchronized by bleaching with 1.3% NaOCl and 0.5 M KOH, according to methods described by Porta-de-la-Riva et al. [49]. Embryo floatation was performed with 60% sucrose-0.1 M NaCl mixture (ratio: 1:1) and subsequently re-suspended in M9 buffer. Hatching into L1 larvae was done on NGM agar plates without bacteria for ~ 20 h to obtain a synchronous L1 population.

Approximately 860 synchronized L1 larvae per 10-cm-diameter OP50 *E. coli* seeded NGM plate were incubated at 20 °C for 76 h to an adult population at maximum reproduction stage (Fig. 1b).

#### *Caenorhabditis elegans* ivermectin exposure

Synchronized N2 adults at maximum reproduction (~ 300 worms/ml) were initially incubated in S-complete media supplemented with 10^–9^, 10^–11^, 10^–13^ M IVM (+ 0.025% DMSO final concentration), and media^+DMSO^ (control). However, because the above-mentioned IVM concentrations had no discernible effect on the worms' behavior, the concentrations were increased to 10^–7^, 10^–8^, 10^–9^ M IVM (+ 0.025% DMSO final concentration). All treatments were set up in biological quadruplets supplemented with 1 mg/ml OP50 *E. coli* and incubated for 4 h at 20 °C (Fig. 1b).

### Behavioral assays

After 4 h incubation at 20 °C, worms were phenotypically scored for each IVM concentration and control at room temperature. Scoring was based on three assays and performed with minor modifications as previously described by Johnson et al. [50].

The pharyngeal pumping assay was performed on three randomly selected worms per biological replicate. Worms were placed on 6-cm-diameter NGM plate containing a lawn of OP50 *E. coli*. Under an 80× magnification stereomicroscope (Nikon, Amstelveen, The Netherlands), the number of pharyngeal pumps per minute for each worm was counted.

The thrashing assay was performed on 12 worms, 3 randomly selected from a pool of at least 6 worms per biological replicate. Scoring was performed within their respective treatment solutions. Worms were video recorded for at least a minute. For scoring, a photographic image of the pool was taken at 0 s. Worms were randomly assigned numbers between one and *n*, where *n* is the total number of worms in the pool. Assigned numbers were randomized thrice, and three numbers were drawn. Worms with the drawn number were used for the scoring. The number of thrashings per minute was recorded. A thrash was defined as a complete sinusoidal motion from maximum to minimum amplitude and back.

In the dispersal assay, three to five worms from each treatment group were placed on the opposite edge of an OP50 lawn on a 6-cm-diameter NGM plate. Plates were scored after ~ 35 min based on the percentage of worms that left the inoculation spot and reached the OP50 lawn.

### *Caenorhabditis elegans* RNA extraction, quality check, and cDNA synthesis

The remaining approximately 300 *C. elegans* worms were retrieved from the wells, washed twice in ice-cold M9 buffer, and centrifuged to pellet. The pellet was frozen in liquid nitrogen, ground with a pestle, and suspended in 900 μl Trizol (Invitrogen, Carlsbad, CA, USA) during continuous grinding. The aqueous phase of the homogenized suspension was isolated using chloroform and then advanced into the NucleoSpin® RNA Plus Kit (Macherey Nagel, Düren, Germany) for RNA extraction according to the manufacturer's instructions. RNA quality and quantity checks were performed on TapeStation 4150 (Agilent, Santa Clara, CA, USA) according to manufacturer’s instructions. Two micrograms of total RNA was reverse transcribed using SuperScript™ III First-Strand Synthesis System (Invitrogen, Carlsbad, CA, USA) to generate cDNA, which was then used in the qPCR assays.

### Primer design and optimization

Coding sequences (CDS) for the candidate genes were retrieved from the *P. univalens* genome (accession no. PRJNA386823) in the WormBase ParaSite database [51]. Primers were designed on an open-source online application, primer3 version 0.4.0, available at http://bioinfo.ut.ee/primer3-0.4.0/, aiming for an amplicon size of approximately 500 bp, spanning at least two exons (Additional file 4: Table S1). For genes with multiple transcripts, only CDSs for transcripts with the highest read counts in the RNA sequencing (RNAseq) data were chosen. Coding sequences were aligned in CodonCode Aligner program (version 9.0.1, CodonCode Corporation) and unique or homologous regions selected for the design of primers. Polymerase chain reaction (PCR) was performed on a thermocycler (Applied Biosystems, Waltham, MA, USA) in a reaction mix consisting of 12.5 μl ToughMix (QuantaBio, Beverly, USA), 1 μl each 10-μM primer, 9.5 μl water, and 1 μl cDNA as a template, synthesized from 1 µg *P. univalens* RNA extract. The PCR program was as follows: initial denaturation at 95 °C for 5 min, 40 cycles of denaturation at 95 °C for 45 s, 55 °C annealing temperature for 45 s, elongation at 72 °C for 1 min, and final elongation at 72 °C for 5 min. Amplicons were submitted for Sanger sequencing at Macrogen (Amsterdam, The Netherlands). Resulting sequences were quality assessed and aligned to obtain consensus sequences in CodonCode Aligner. As a validation step, BLAST searches of consensus sequences against their corresponding CDS were performed using WormBase ParaSite. Thereafter, consensus sequences were used to design Reverse Transcriptase-qPCR (RT-qPCR) primers (Additional file 4: Table S1) using the same online platform as above, aiming for an amplicon size of 75–150 bp.

Similarly, CDS of *C. elegans* orthologues were retrieved from WormBase [52] using respective gene IDs. For orthologues with splice variants, primers were designed for CDS regions that were homologous to all variants. Likewise, amplicons were Sanger sequenced at Macrogen (Amsterdam, The Netherlands) and validated by BLAST searches against their corresponding CDS in the WormBase database. Reverse Transcriptase-qPCR assays were optimized for primer concentration, annealing temperature, and efficiency (Additional file 4: Table S1).

### Relative gene expression of candidate genes using reverse transcriptase qPCR

Samples and non-template controls were run in duplicate reactions of 25 μl, composed of 12.5 μl Quantitect SYBR Green PCR mix (Qiagen, Hilden, Germany), 1 μl of each 10-μM primer, 8.5 μl water, and 2 μl (dilution factor: 5) cDNA as template on a CFX96 Touch PCR machine (Bio-Rad, Solna, Sweden). The RT-qPCR program was as follows: initial denaturation at 95 °C for 15 min, 40 cycles of denaturation at 95 °C for 15 s, assay specific annealing temperature for 30 s, and elongation at 72 °C for 30 s. For amplicon verification, a subsequent melt curve analysis was performed by temperature elevation from 60 to 95 °C, with increments of 0.5 °C for 5 s.

The Livak method [53] was used to determine the relative expression of the candidate genes. The gene expression was normalized to the geometric mean of two references genes for each species, i.e. actin, *act-5* (PgR070_g023) and flyceraldehyde-3-phosphate dehydrogenase, *gpd-1* (PgB20_g009) for *P. univalens*, and *tba-1* (tubulin, alpha) and *eif-3.C* (eukaryotic initiation factor) for *C. elegans* and related to media^+DMSO^ controls (Additional file 5: Table S2 and Additional file 6: Table S3). Gene expression was presented as log_2_ fold change. As a validation step, the log_2_ fold change values of candidate genes from RNAseq DEG analysis and RT-qPCR assays were imported into R and graphically compared in gglpot2’s scatterplot.

### Statistical analysis

The average counts of behavioral assays were calculated in Microsoft Excel (Additional file 7: Table S4) and then imported into GraphPad Prism [Version 9.1.0 (221)] for statistical analysis and visualization. In a one-way ANOVA with Dunnett’s multiple comparisons test, average counts for the drug-treated worms were compared to media^+DMSO^ controls and graphically represented.

Log_2_ fold changes of gene expression in the IVM exposed biological replicates were imported into GraphPad Prism [version 9.1.0 (221)] for each candidate gene and graphically represented. Graphical data are presented as mean values with a standard error of the mean (SEM) where applicable.

## Results

### *Parascaris univalens* RNAseq and differential gene expression

Adult *P. univalens* worms, collected from Icelandic horses, were treated with sublethal doses of IVM in vitro (10^–13^, 10^–11^, 10^–9^ M IVM for 24 h), and non-treated worms served as controls (see “Materials and methods”). Total RNA with RIN values ranging from 6.6 to 8.7 was sequenced on an Illumina Nova Seq. After read quality control with fastp, the number of reads per sample ranged between 63 and 132 million. Read sequences are available at https://www.ebi.ac.uk/ena [European Nucleotide Archive (ENA), accession no. PRJEB37010]. Read mapping against the reference transcriptome (https://parasite.wormbase.org/Parascaris_univalens_prjna386823/Info/Index/) yielded an average rate of 80–90% mapped reads, indicative of a high similarity between this transcriptomic data and reference transcriptome.

**Fig. 2:**
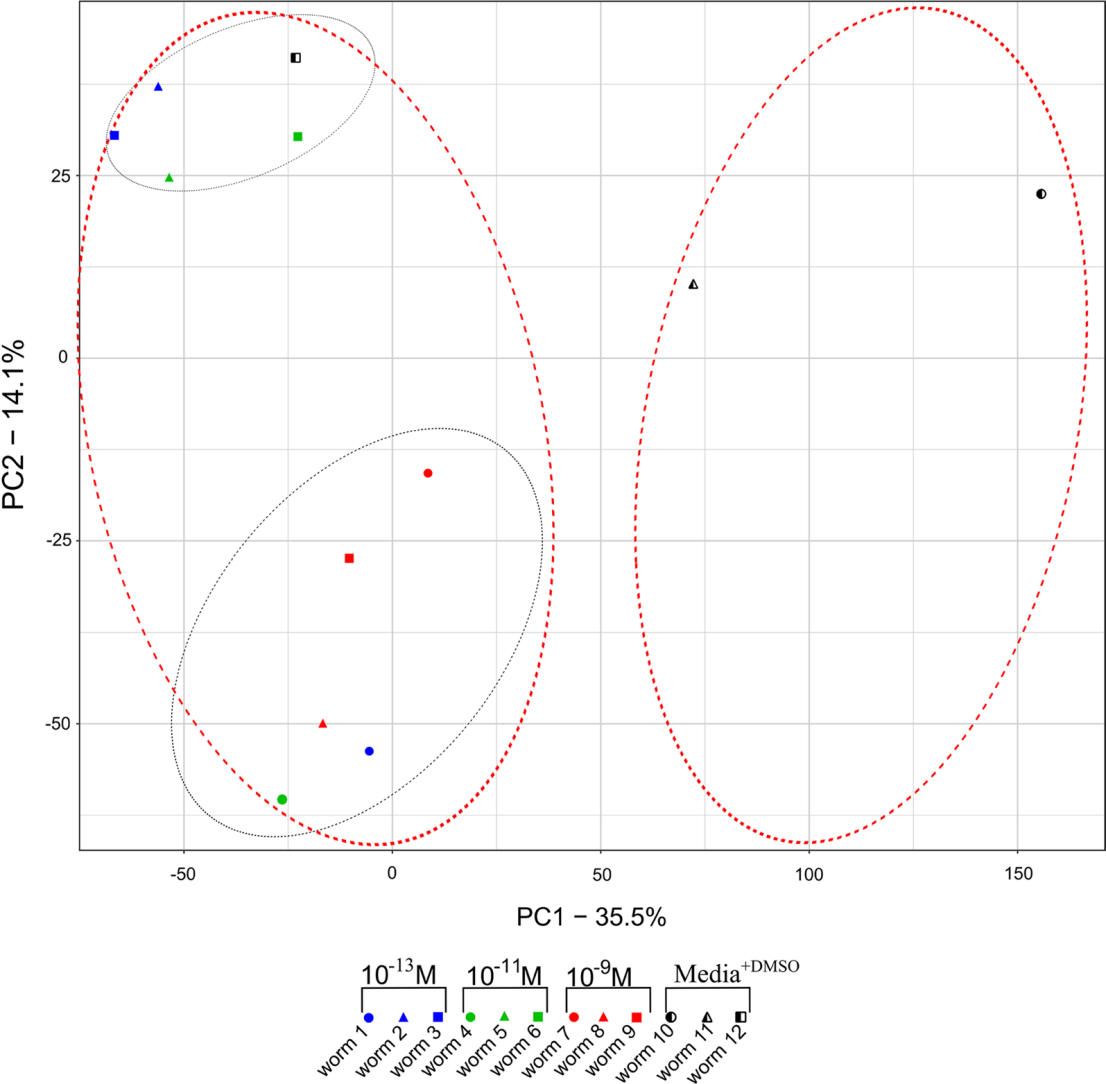
Principal component analysis plot of *Parascaris univalens* based on scaled read counts of 10,830 genes showing variance among individual worms after exposure to three IVM drug concentrations (10^−13^ M, 10^−11^ M and 10^−9^ M) and media^+DMSO^ (control). PC1 and PC2 represent the largest variances in the data, further portrayed by red and black dotted ellipses, respectively

After PCA, 12 PCs were revealed of which PC1 and PC2 accounted for the most variation in the data, 35.5% and 14.1%, respectively (Fig. 2 and Additional file 1: Fig. S1). PC1 grouped worms into IVM treated and IVM untreated, except for worm 12. However, PC2 revealed variation in response within biological replicates but worms 1, 4 and 12 appeared away from their treatment groups (Fig. 2). Although effort was taken to select only females for the experiment, individual 2 in 10^–11^ M lacked an egg-containing uterus upon dissection. However, because this individual did not appear to be an outlier in the PCA plot (Fig. 2), it was included in the analysis.

Following DESeq2 analysis of the 1085 genes (> 0.5-fold, adj. *P*-value < 0.05) differentially expressed across the three drug concentrations, 65% (703 genes) had known functions according to Swiss-Prot annotations. Twenty-one percent of the DEGs were found in 10^–13^ M, 17% in 10^–11^ M, and 62% in 10^–9^ M (Additional file 2: Fig. S2). The overall amplitude of gene expression changes after IVM treatment was small; 84 genes were upregulated more than twofold and 147 genes were downregulated more than twofold. There was also a quite high level of variability in gene expression between the different worms.

Selection of candidate genes for further characterization was based on the presence of an orthologue in *C. elegans*, level of differential gene expression and functional characterization as drug target or involvement in at least one of the following processes: xenobiotic metabolism, metabolite transportation, or gene expression regulation. Fifteen candidate genes were selected (11 up- and 4 downregulated) and further categorized into six groups: drug target, phase I metabolic enzyme, phase II metabolic enzyme, transporter, transcription regulator, and others (Table 1). The I-TASSER web server was used to verify *P. univalens*–*C. elegans* similarity for 80% and 87% of the candidate genes based on molecular function and biological process, respectively (Table 1).

**Table 1:** I-TASSER web server comparison of selected candidate genes’ orthologues in *Caenorhabditis elegans* and *Parascaris univalens* (geneIDs begin with “Pg”)

	GeneID	GO:MF^a^	GO-score^c^	GO:BP^b^	GO-score	*P. univalens* RNAseq log_2_ fold change		
						10^–13^ M	10^–11^ M	10^–9^ M
Drug target	*lgc-37*	GO:0004889	0.34	GO:0006811	0.73			8.20
	*PgR047_g061*		0.33		0.60			
Phase I enzyme	*dhs-2*	GO:0005488	0.83	GO:0055114	0.83	1.40	1.50	1.50
	*PgR127_g021*	GO:0004303	0.36		0.87			
	*dhs-4*	GO:0004316	0.73	GO:0055114	0.81		1.90	
	*PgR004_g112*	GO:0005488	0.83		0.83			
	*dhs-27*	GO:0004316	0.81	GO:0055114	0.81	1.80	1.70	1.90
	*PgR007_g080*		0.59		0.77			
	*F13D11.4*	GO:0050662	0.98	GO:0055114	0.80	− 1.50	− 1.60	− 1.10
	*PgB01_g106*		0.97		0.76			
Phase II enzyme	*C13A2.7*	GO:0008171	0.52	GO:0032259	0.35	1.20		1.90
	*PgR018_g071*	GO:0008376	0.53	GO:0043413	0.56			
	*ugt-54*	GO:0016758	0.70	GO:0030259	0.52	− 0.80		− 1.20
	*PgB20_g050*		0.72		0.62			
Transport	*cup-4*	GO:0004889	0.45	GO:0006811	0.65	− 2.80		− 2.10
	*PgR075_g041*		0.48		0.67			
	*hmit-1.2*	GO:0015168	0.36	GO:0055085	0.40	2.40	2.10	2.30
	*PgR015_g078*	GO:0042900	0.32		0.38			
	*hmit-1.3*	GO:0042900	0.36	GO:0055085	0.41	2.40	2.10	2.30
	*PgR015_g078*		0.32		0.38			
	*slc-17.2*	GO:0042900	0.57	GO:0055085	0.57	− 1.00		− 1.10
	*PgR047_g023*		0.57		0.61			
	*Y71G12B.25*	GO:0015168	0.55	GO:0055085	0.69	1.60		1.50
	*PgR005X_g127*		0.54		0.64			
	*F17C11.12*	GO:0042900	0.34	GO:0055085	0.51	− 1.00		0.70
	*PgR003_g012*		0.51		0.39			
Transcription regulator	*nhr-3*	GO:0003700	0.65	GO:0006355	0.65	1.2	0.90	
	*PgR005X_g204*		0.50		0.50			
Others	*F08F8.7*	GO:0004750	1.00	GO:0006098	0.94	8.90		
	*PgR013_g129*		0.99		0.67			
	*pkc-1*	GO:0005524	0.60	GO:0014059	0.32		1.00	0.90
	*PgB05_g097*		0.64		0.31			

^a^GO term: molecular function

^b^GO term: biological process

^c^Average weight assigned to the GO term, where the weights are determined using a combined measure of global and local similarity between the query and template proteins. It is a numeric value between 0 and 1, with higher values indicating more certain predictions

### *Caenorhabditis elegans* behavior assays after IVM exposure

Synchronized *C. elegans* N2 adults were initially exposed to similar IVM concentrations as adult *P. univalens* (10^–9^, 10^–11^, 10^–13^ M). However, as there were no observable phenotypic or gene expression effects on *C. elegans* with such low IVM concentrations (data not shown), the drug concentrations were increased to 10^–9^, 10^–8^, 10^–7^ M, respectively.

**Fig. 3:**
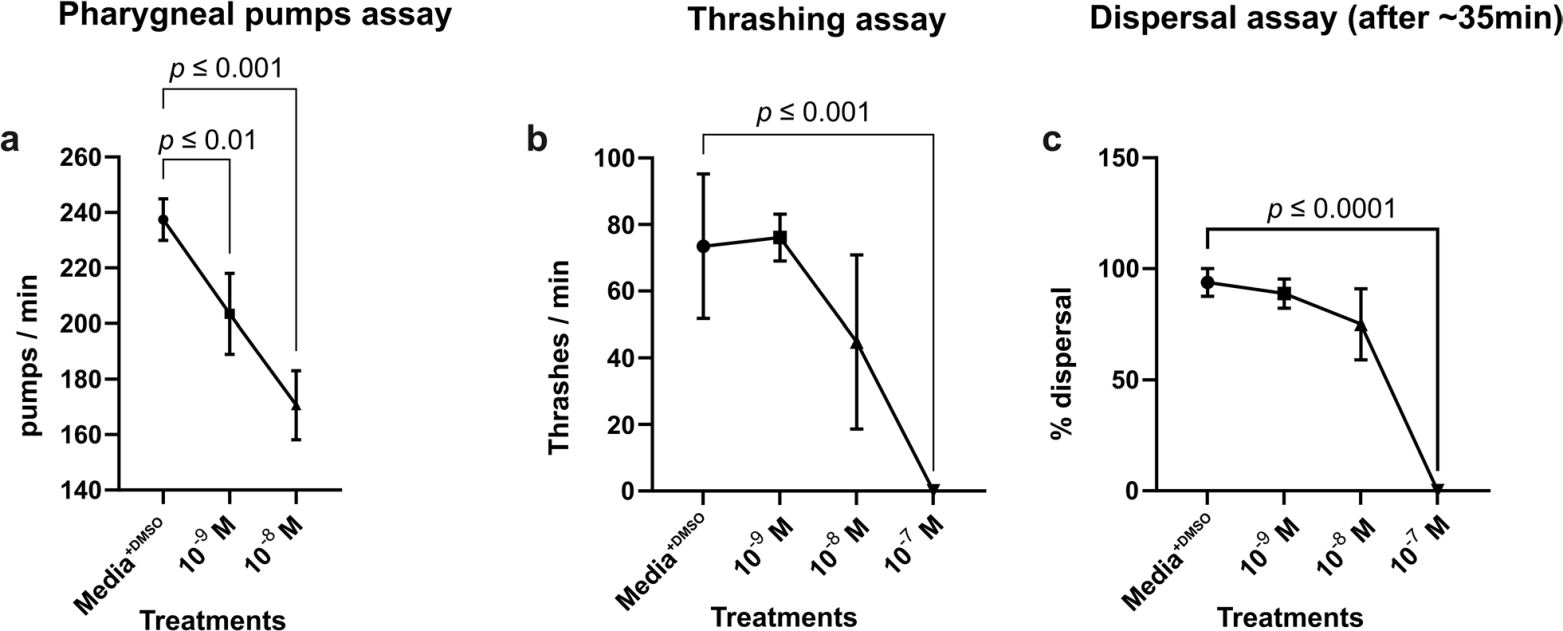
Behavioral assays for *Caenorhabditis elegans*. **a** Pharyngeal pumps per minute, **b** body thrashes per minute, and **c** dispersal after IVM exposure, 10^–9^ 10^–8^, 10^–7^ M, and control (media^+DMSO^). Mean number (± standard error, SE) of viability scores in treatments were compared to those of the control in a one-way ANOVA (Dunnett’s multiple comparisons test) to elucidate significant differences

Increased IVM concentration showed an observable effect on the worms in all behavioral assays (Fig. 3). Ivermectin exposure also showed a significant dose-dependent reduction of the number of pharyngeal pumps per minute (Fig. 3a). In the thrashing assay, IVM concentrations (10^–8^ and 10^–7^ M) significantly reduced thrashes, with even immobile worms in the 10^–7^ M concentration demonstrated by the very low adjusted *P*-value (*P* ≤ 0.001) (Fig. 3b). The dispersal assay showed a similar dose-dependent trend, but was significant only in 10^–7^ M IVM (Fig. 3c). Worms incubated in media^+DMSO^ (control) showed no observable effects and no significant behavioral differences (Fig. 3). The 10^–7^ M concentration was excluded from the qPCR assays, as we aimed for mechanisms at play only in sub-lethal conditions.

**Fig. 4:**
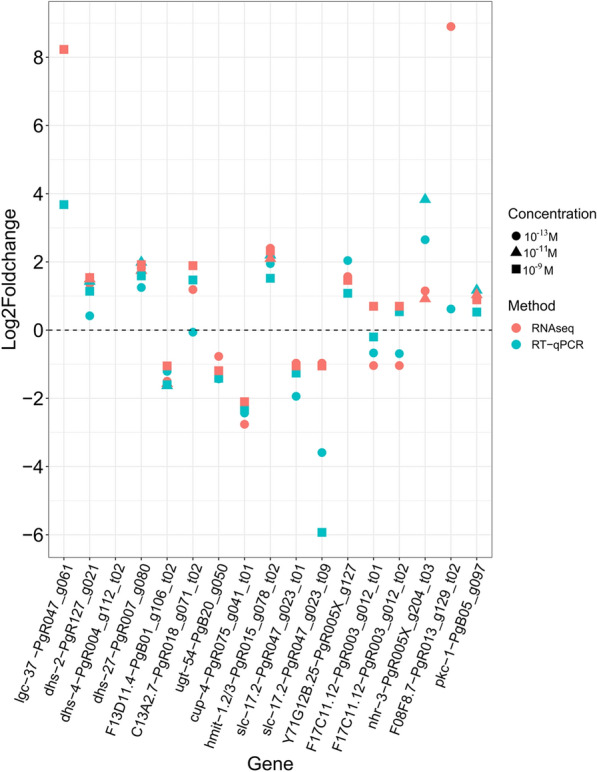
Scatter plot comparing relative gene expression (log_2_ fold change) of *Parascaris univalens* candidate genes between two methods [ RNAseq (orange) and RT-qPCR (blue)] after exposure to 10^–13^, 10^–11^, and 10^–9^ M IVM for 24 h

### Expression of candidate genes in *P. univalens* and *C. elegans* after IVM exposure

Log_2_ fold change of candidate genes from RNAseq DEG analysis appeared consistent to those from RT-qPCR, with very few exceptions (Fig. 4). To verify differential gene expression in IVM-treated *P. univalens* and *C. elegans*, RNA with RIN values ranging from 6.6 to 9.8 were used in RT-qPCR assays, and all gene expressions described below are based on these assays. For simplicity, only *C. elegans* gene names are used for both *P. univalens* and *C. elegans*. Corresponding *P. univalens* gene names can be referenced in Table 1,1.

**Fig. 5:**
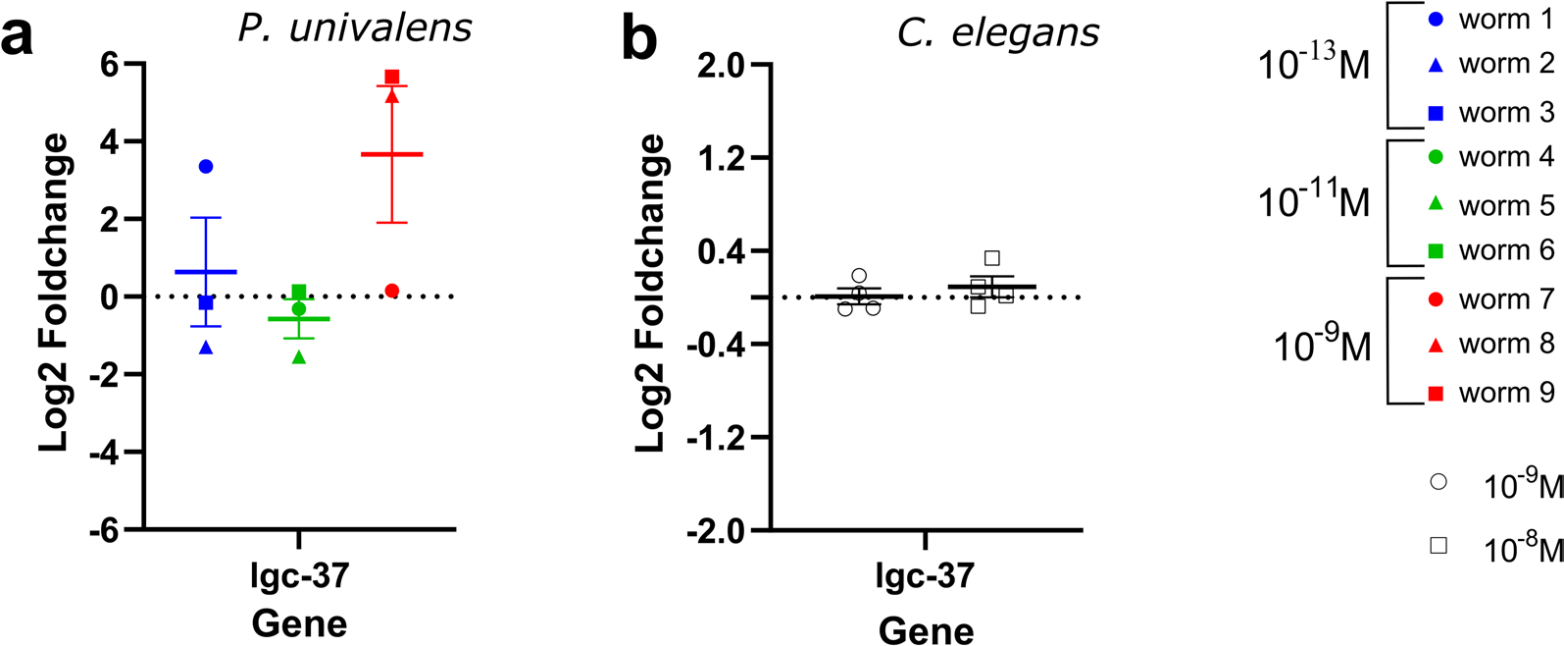
Relative gene expression (log_2_ fold change) of the GABA receptor subunit *lgc-37* in **a** *Parascaris univalens* after exposure to 10^–13^, 10^–11^, and 10^–9^ M IVM for 24 h and **b** *Caenorhabditis elegans* after exposure to 10^–9^ and 10^–8^ M IVM for 4 h

**Fig. 6:**
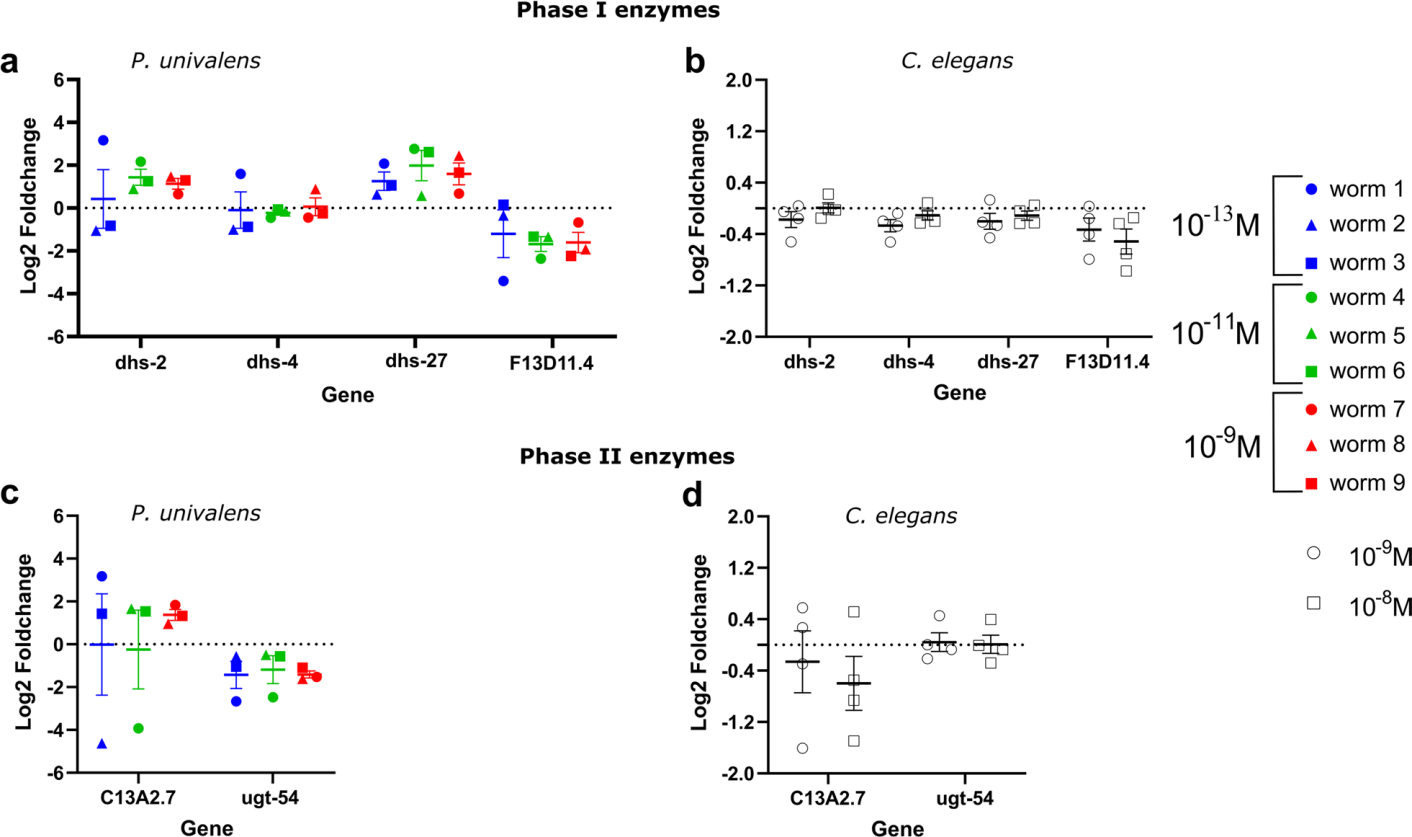
Relative gene expression (log_2_ fold change) of Phase I and II metabolic genes in *Parascaris univalens* (**a**, **c**) after exposure to 10^–13^, 10^–11^, and 10^–9^ M IVM for 24 h. Relative gene expression (log_2_ fold change) of Phase I and II metabolic genes in *Caenorhabditis elegans* (**b**, **d**) after exposure to 10^–9^ and 10^–8^ M IVM for 4 h

**Fig. 7:**
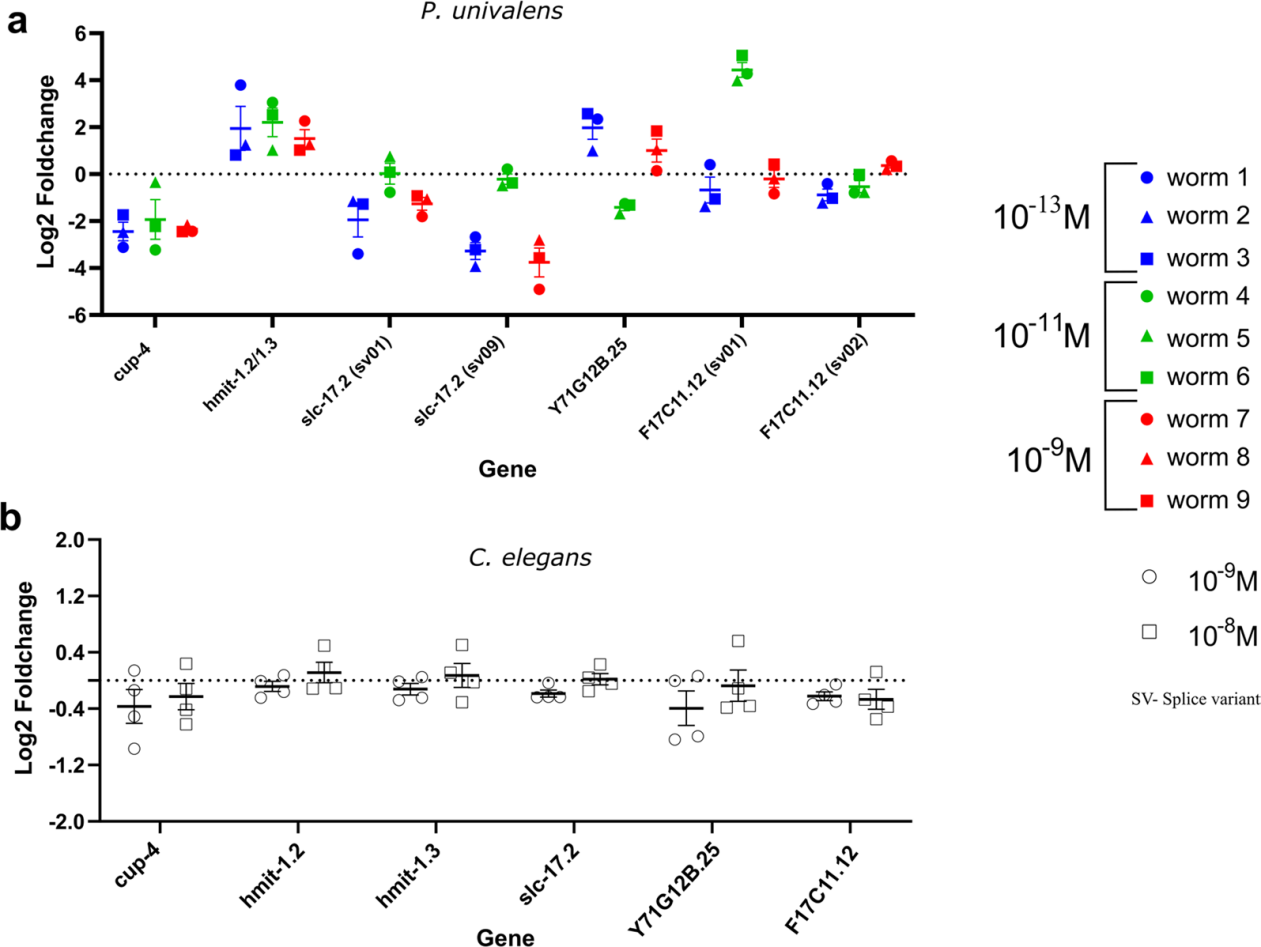
Relative gene expression (log_2_ fold change) of transport protein genes in *Parascaris univalens* **a** after exposure to 10^–13^, 10^–11^, and 10^–9^ M IVM for 24 h. Relative gene expression (log_2_ fold change) of transport protein genes in *Caenorhabditis elegans* **b** after exposure to 10^–9^ and 10^–8^ M IVM for 4 h

**Fig. 8:**
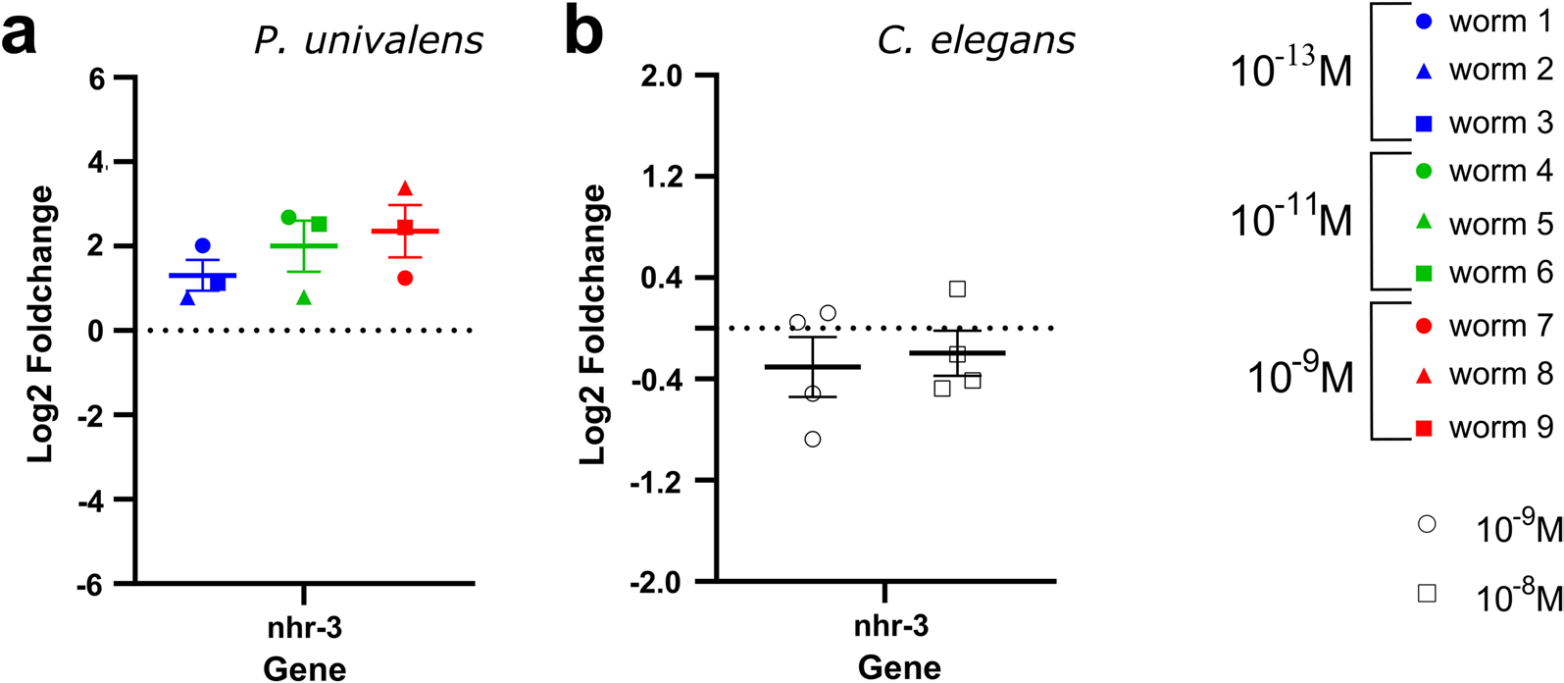
Relative gene expression (log_2_ fold change) of *nhr-3* transcription regulator in *Parascaris univalens* **a** after exposure to 10^–13^, 10^–11^, and 10^–9^ M IVM for 24 h. Relative gene expression (log_2_ fold change) of *nhr-3* transcription regulator in *Caenorhabditis elegans* **b** after exposure to 10^–9^ and 10^–8^ M IVM for 4 h

In general, although IVM appears to have an effect on the expression levels of candidate genes in *P. univalens*, this effect appears to vary among individual worms, agreeing with the observations seen in the RNAseq-based PCA plot (Fig. 2). In *C. elegans*, however, even if there is a strong phenotypic effect of IVM, it has marginal to no effect on the change in expression levels of any of the 15 selected candidate genes (Figs. 5, 6, 7, 8).

The GABA receptor subunit gene, *lgc-37*, a putative drug target for IVM, showed a generally increased trend in change in expression in *P. univalens* after IVM exposure (Fig. 5a); however, increments in *C. elegans* were marginal (Fig. 5b).

The change in expression profiles of the Phase I metabolic genes in *P. univalens* showed non-uniform patterns. The short chain dehydrogenases (SDR) *dhs-2* and *dhs-27* showed an increased change in expression while *F13D11.4* was decreased in *P. univalens* (Fig. 6a). The expression change of Phase II metabolic genes varied. The glucuronosyltransferase gene *ugt-54* was decreased in *P. univalens*, while transferase *C13A2.7* showed a trend of increased change in expression (Fig. 6c). However, in *C. elegans*, all genes generally appear not to be affected by the IVM exposure (Fig. 6b, d).

All investigated transport protein genes were enriched with major facilitator superfamily (MFS) members, except *cup-4*, which belongs to the ligand-gated ion channel family. Overall change in expression of transport protein genes showed a decreased trend in *P. univalens* and minor to no change in *C. elegans* (Fig. 7). Genes *hmit-1.2/hmit-1.3* and *Y17G128B.25* showed an increased change in expression in *P. univalens* (Fig. 7a).

Finally, in response to IVM, transcriptional regulator, *nhr-3*, showed an increased change in gene expression trend in *P. univalens* (Fig. 8a) but marginal to no change in expression was observed in *C. elegans* (Fig. 8b).

The neural signaling protein, *pkc-1*, showed an increased change in gene expression in *P. univalens* exposed to 10^–11^ and 10^–9^ M IVM (Additional file 3: Fig. S3A), but showed little to no change was observed in *C. elegans* (Additional file 3: Fig. S3B). Only the 10^–13^ M IVM showed marginal increased change in expression of ribulose-phosphate 3-epimerase gene *F08F8.7* in *P. univalens*, and little to no change in *C. elegans* across all IVM concentration was observed in both species (Additional file 3: Fig. S3).

## Discussion

Recent studies show that the equine roundworm *P. univalens* has developed resistance to the three major classes of anthelmintic drugs used and that the emergence of multidrug-resistant variants is apparent [4, 12, 54]. While the cause of drug resistance drug in parasites is poorly understood, the primary resistance mechanism to benzimidazoles has been better explored than those of other drug classes, particularly in clade V nematodes (see [15] for review). Consequently, most of the proposed resistance mechanisms have been derived from clade V nematodes and may therefore differ in Clade III nematodes such as *P. univalens*. It is critical to have a thorough understanding of *P. univalens* resistance mechanisms to potentiate species-specific interventions for resistance control and/or alleviation. This, however, is largely constrained by the complexity of its host-dependent life cycle and a lack of in vitro experimental models.

In this study, we evaluated the usage of *C. elegans* as a model for *P. univalens* by a comparative gene expression approach. Of the 1085 DEGs in *P. univalens* after IVM exposure, 231 DEGs had a fold change above twofold. This is however not unique to *P. univalens* as another clade III member, *Brugia malayi*, exposed to 10^–7^ M IVM for 24 h, demonstrated similar modest gene expression changes (14 genes upregulated and 8 genes downregulated more than twofold) [55]. While changes in gene expression greater than twofold are generally considered more important, expression changes of up to 0.5-fold have been proposed to be as equally meaningful in RNAseq assays if the false discovery rate is < 5% (adj. *P*-value 0.05), with at least three biological replicates used and data analyzed using the Deseq2 R program [56]. All DEGs in the current study followed the above recommendation and were therefore treated equally. Fifteen DEGs were selected as candidate genes and were further evaluated and compared with orthologous genes in *C. elegans* exposed to IVM.

Although IVM affected gene expression in *P. univalens*, there was little to no response in expression of the candidate genes in *C. elegans*, despite the significant dose-dependent behavioral effect of IVM on adult *C. elegans* worms. The premises for *C. elegans* as a model for research in parasitic nematodes have been thoroughly described in other studies [40, 57–59]. For example, *Ascaris suum* and *C. elegans* share 68.9% predicted genes [60], which supports *C. elegans* as a suitable model for the *Ascarididae* family. However, despite all the above, our results show differences in expression of the selected genes between *C. elegans* and *Parascaris univalens* after exposure to IVM. This observation can be attributed to a variety of factors. *C. elegans* and *P. univalens* are members of different clades, V and III, respectively, and they also have different life cycles and evolutionary histories [61]. As a result, genes and gene families associated with parasitism are either absent or have different functions in *C. elegans* [62–64]. The chosen candidate genes in this study are a small subset (1.4%) of all the genes responding to IVM exposure in *P. univalens*. Therefore, a global transcriptomic approach comparing whole worm transcriptomes of both *C. elegans* and *P. univalens* after IVM exposure may be a better method to evaluate whether *C. elegans* can be an accurate in vitro model for *P. univalens*.

The putative IVM target, *lgc-37* (GABA receptor subunit), showed an increased expression in *P. univalens*, and Martin et al. [29] reported a similar observation. Increased expression of *lgc-37* has not been correlated to AR; however, an amino acid substitution (K169R) in *H. contortus lgc-37* transfected in *C. elegans* has been reported to exhibit reduced sensitivity to ML [65]. Taken together, these findings indicate that *lgc-37* is important and that additional research is necessary to fully understand its role in* P. univalens*’ response to IVM*.*

In *P. univalens*, decreased expression was observed for the genes belonging to MFS-transport proteins *slc-17.2* and *F17C11.12* and the ligand-gated ion channel *cup-4* after exposure to IVM. The role of MFS genes in parasitic worms has yet to be determined, but their involvement in drug resistance in bacteria and yeast has been reported [66]. Other than the efflux role, MFSs mediate cellular uptake of glucose and other saccharides [67]. Because IVM causes worm starvation through pharyngeal paralysis [68–70], we speculate that the decreased glucose concentration, a consequence of starvation, may trigger decreased gene expression of MFS responsible for glucose or other saccharide uptake. The ATP-binding cassette transporters, primarily Pgp efflux pumps, have been implicated in resistance in parasitic nematodes [21, 22, 24]. Interestingly, we found no DEGs of Pgps in our RNAseq dataset, which is consistent with previous studies in *P. univalens* [28, 30, 31, 71]. However, other studies have reported induced gene expression of Pgps in IVM-resistant larvae of *Cooperia oncophora* [72] and multi-drug resistant larvae of *H. contortus* exposed to IVM [22].

Differential expression of several Phase I enzymes was observed in *P. univalens* after IVM exposure. Expressions of *dhs-2* and *dhs-27* were increased whereas the reverse was seen for *dhs-4* and *F13D11.4.* However, in *C. elegans*, all SDR genes showed a consistent minor decrease in expression levels. The role of SDRs in anthelmintic resistance is not well known. However, in several helminths, SDR genes have been shown to be involved in metabolic activities of BZs [73–77]. Even though we did not observe any major expressional response of SDR genes in *C. elegans* after IVM exposure, an increase in expression of *dhs-23* in a BZ-resistant strain of *C. elegans* after in vitro exposure to BZ derivatives has been reported [35].

Expression of the Phase II enzyme, *ugt-54*, was decreased in *P. univalens* after IVM exposure, but no response was observed in *C. elegans*. Similarly, decreased expression of other members of the UGT family, *ugt-3*, *ugt3a1*, *ugt-47*, and *ugt-48*, have been observed in *P. univalens *in vitro exposed to pyrantel citrate, thiabendazole, and oxibendazole [29, 30]. Although we did not observe any response of *ugt-54* in *C. elegans*, other studies have shown differential gene expression of UGTs in IVM-tolerant strain of *C. elegans* after exposure to 10^–6^ M IVM [78] and in the BZ-resistant strain of *C. elegans* after exposure to BZs [35, 79, 80]. Together, it is clear that UGTs are important in drug metabolism in both nematode species, and their role in anthelmintic resistance in *P. univalens* needs to be further elucidated.

The transcriptional regulator, *nhr-3*, showed increased change in expression in *P. univalens* after IVM exposure but a slightly decreased change in *C. elegans*. Increased expression of *nhr-8* has reported to upregulate expression of Pgps and Phase I enzyme genes in *C. elegans* and resulted in a decreased efficacy of IVM [81]. A similar effect of IVM has also been observed in *C. elegans* carrying a transgenic construct expressing *H. contortus-nhr-8* [81], suggesting that the function of *nhr-8* could be similar in different nematode species. Even though *nhr-3* does not respond in a similar way in *C. elegans* and *P. univalens*, the NHR family may play a role in AR.

## Conclusion

In summary, this is the first time *C. elegans* has been evaluated as a model for *P. univalens* using a comparative gene expression approach. However, the genes selected showed dissimilar expression patterns between the two nematode species. This difference in expression could be due to caveats in the experimental design or simply the differences in life cycles of *P. univalens* and *C. elegans*. For example, due to the difficulties in obtaining live adult *P. univalens*, only three individuals were used per biological replicate in the study, compared to 300 worms for *C. elegans*. In addition, the unnatural in vitro culture environment may influence undesired behavioral changes or gene expression in *P. univalens* worms [29]. Furthermore, only 1.4% of RNAseq-derived DEGs from *P. univalens* were used as candidate genes in this study, of which some transcripts (splice variants) also showed varying expression patterns. Together, this limits the ability to draw firm conclusions regarding comparability between these two nematode species. However, with comparative genomics, using RNAseq data from IVM exposed *C. elegans* and *P. univalens*, it is plausible to find potential drug targets with similar expression in both nematodes. This could provide a better understanding of drug response mechanisms to further investigate and combat drug resistance.

## Supplementary Information

**Additional file 1: Figure S1.** A screeplot showing variation explained by all principal components.

**Additional file 2: Figure S2.** Venn diagrams showing the number of differentially expressed genes in *P. univalens* that are shared among three IVM concentrations 10^–13^, 10^–11^, and 10^–9^ M. Genes with an adjusted *P*-value (Walds test and Benjamini-Hochberg procedure) < 0.05 were considered differentially expressed.

**Additional file 3: Figure S3.** Relative gene expression (log_2_ fold change) of *pkc-1* neural signaling protein and F08F8.7 Ribulose-phosphate-3-epimerase in *Parascaris univalens* (**a**) after exposure to 10^–13^, 10^–11^ and 10^–9^ M IVM for 24 h. Relative gene expression (log_2_ fold change) of *pkc-1* neural signaling protein and *F08F8.7* ribulose-phosphate-3-epimerase in *Caenorhabditis elegans* (**b**) after exposure to 10^–9^ and 10^–8^ M IVM for 4 h.

**Additional file 4: Table S1.** *Parascaris univalens* and *Caenorhabditis elegans* primer sequences used in PCR assays as well as their respective amplicon sizes, annealing temperatures, and primer efficiencies.

**Additional file 5: Table S2.** *Parascaris univalens* RT-qPCR cycle time values and log_2_ fold change calculations. IVM-13, IVM-11, IVM-9, and CD represent treatment conditions 10^–13^, 10^–11^, 10^–9^ M IVM, and media^+DMSO^ (control), respectively.

**Additional file 6: Table S3.** *Caenorhabditis elegans* RT-qPCR cycle time values and log_2_ fold change calculations. IVM-9, IVM-8, IVM-7, and IVM-D represent treatment conditions 10^–9^, 10^–8^, 10^–7^ M IVM, and media^+DMSO^ (control), respectively.

**Additional file 7: Table S4.** Behavioral assays of *Caenorhabditis elegans* after exposure to 10^–9^, 10^–8^, 10^–7^ M IVM and media^+DMSO^ (control) for 4 h.

## Data Availability

The pipeline used for quality assessment, mapping, and quantification is available (with nextflow and docker support) at https://github.com/SLUBioinformaticsInfrastructure/RNAseq_nf. Nucleotide sequence data reported in this paper are available in the European Nucleotide Archive (ENA) under the accession number PRJEB37010 (https://www.ebi.ac.uk/ena).

## Abbreviations

IVM: Ivermectin
BZ: Benzimindzoles
THP: Tetrahydropyrimidines
ML: Macrocyclic lactones
GluCls: Glutamate-gated ion channels
DEGs: Differentially expressed genes
DMSO: Dimethyl sulfoxide
PCA: Principal component analysis
PC: Principal component
ID: Identification
NaOCl: Sodium hypochlorite
KOH: Potassium hydroxide
NaCl: Sodium chloride
NGM: Nematode growth media
CDS: Coding sequences
PCR: Polymerase chain reaction
RT-qPCR: Reverse transcriptase quantitative polymerase chain reaction
ANOVA: Analysis of variance
SEM: Standard error of the mean
RIN: RNA integrity number
GABA: γ-Aminobutyric acid
SDR: Short chain dehydrogenases
MFS: Major facilitator superfamily
Pgp: P-glycoprotein
