# Supplementary figures and images for: Ivermectin-induced gene expression changes in adult Parascaris univalens and Caenorhabditis elegans: a comparative approach to study anthelminthic metabolism and resistance in vitro
Source: Parasit Vectors. 2022 May 5;15:158. doi: 10.1186/s13071-022-05260-4 (PMC9074254; doi:10.1186/s13071-022-05260-4)

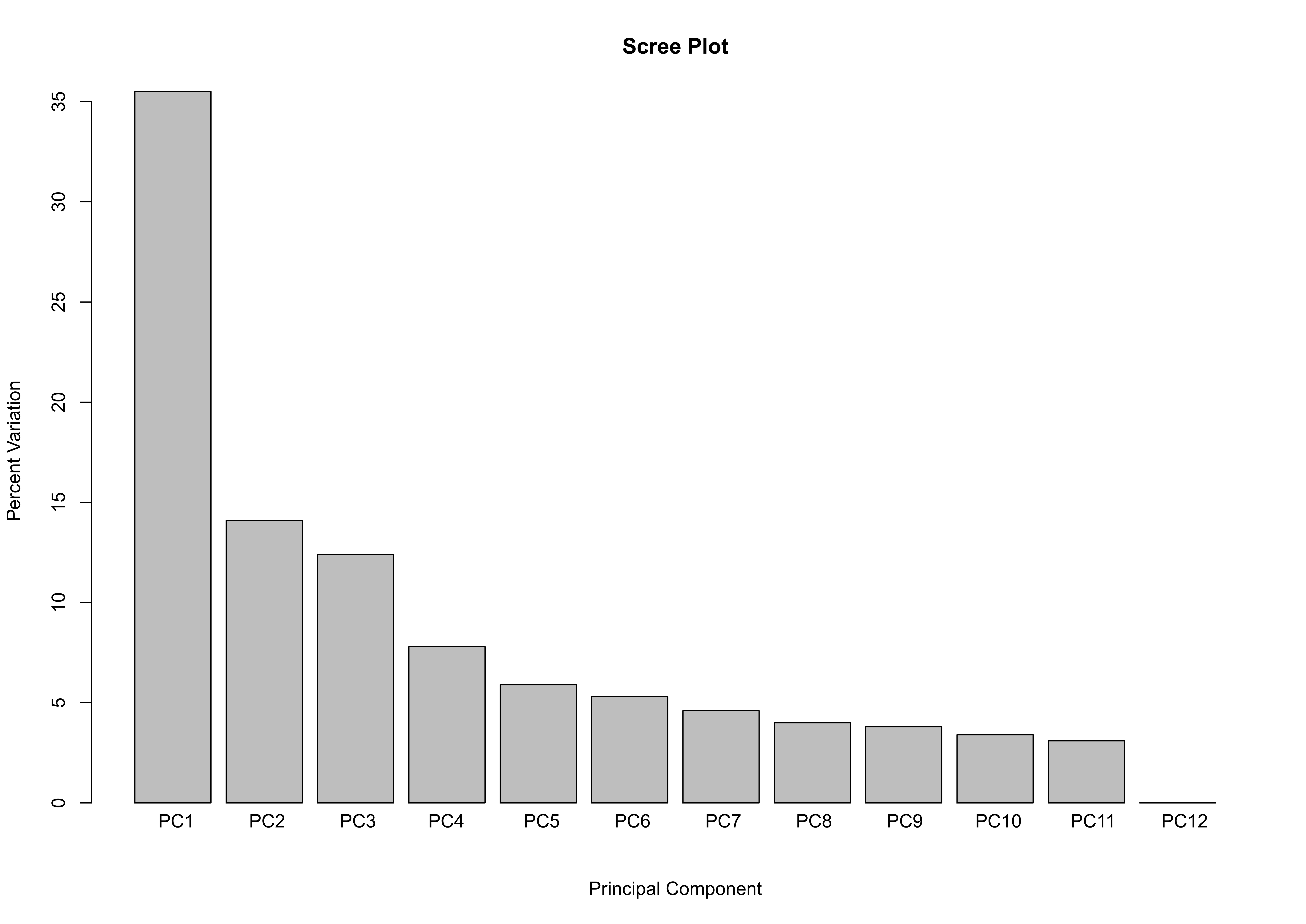

Supplement: Supplementary file 1 — Additional file 1: Figure S1. A screeplot showing variation explained by all principal components. [file 13071_2022_5260_MOESM1_ESM.png]

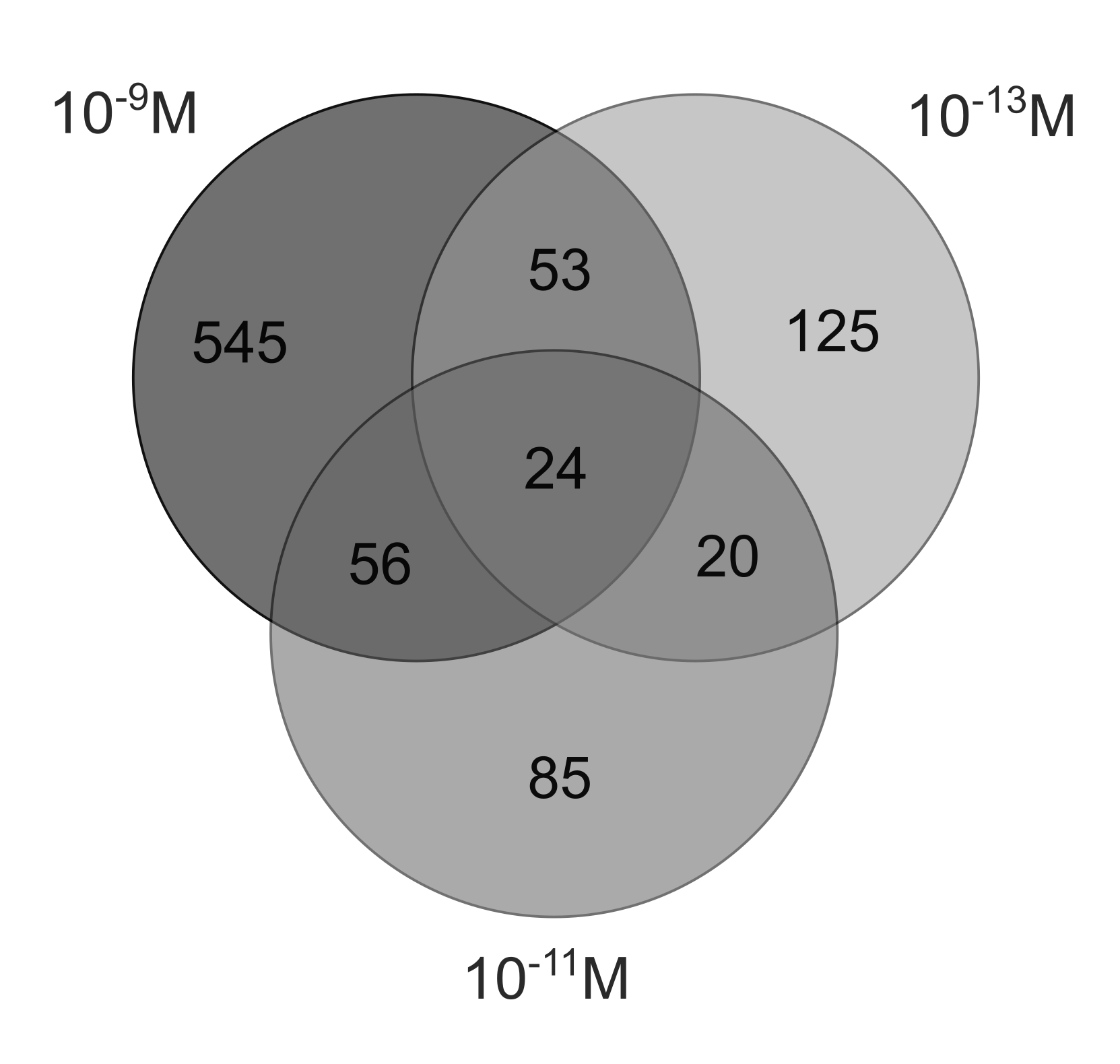

Supplement: Supplementary file 2 — Additional file 2: Figure S2. Venn diagrams showing the number of differentially expressed genes in P. univalens that are shared among three IVM concentrations 10–13, 10–11, and 10–9 M. Genes with an adjusted P-value (Walds test and Benjamini-Hochberg procedure) < 0.05 were considered differentially expressed. [file 13071_2022_5260_MOESM2_ESM.png]

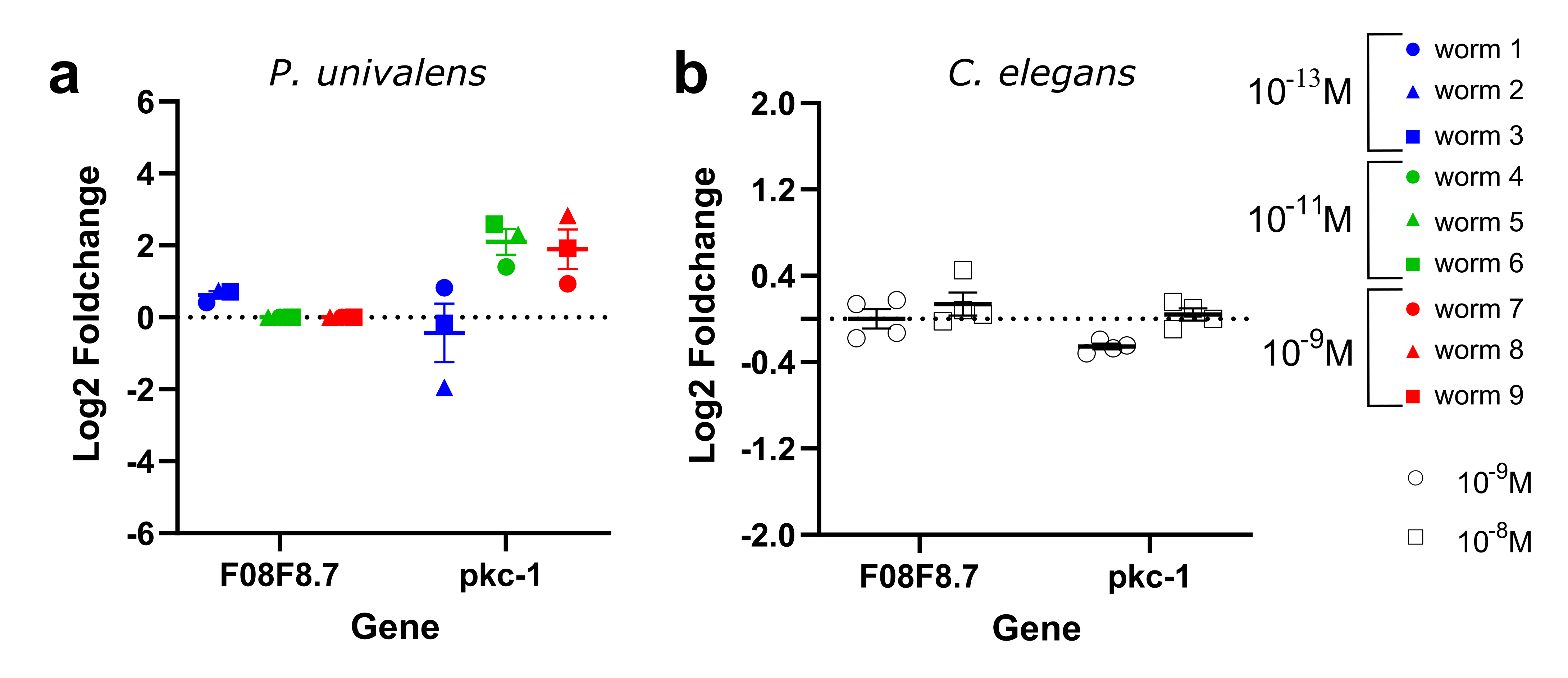

Supplement: Supplementary file 3 — Additional file 3: Figure S3. Relative gene expression (log2 fold change) of pkc-1 neural signaling protein and F08F8.7 Ribulose-phosphate-3-epimerase in Parascaris univalens (a) after exposure to 10–13, 10–11 and 10–9 M IVM for 24 h. Relative gene expression (log2 fold change) of pkc-1 neural signaling protein and F08F8.7 ribulose-phosphate-3-epimerase in Caenorhabditis elegans (b) after exposure to 10–9 and 10–8 M IVM for 4 h. [file 13071_2022_5260_MOESM3_ESM.png]
